# Multisensory learning between odor and sound enhances beta oscillations

**DOI:** 10.1038/s41598-019-47503-y

**Published:** 2019-08-02

**Authors:** A. Gnaedinger, H. Gurden, B. Gourévitch, C. Martin

**Affiliations:** 1 IMNC UMR 8165, Université Paris Sud, Université Paris Saclay CNRS Orsay, Orsay, F-91405 France; 2 Unité de Biologie Fonctionnelle et Adaptative, UMR 8251, Université Paris Diderot, Sorbonne Paris Cité, CNRS, F-75205 Paris, France; 3 Unité de de Génétique et Physiologie de l’Audition, UMRS 1120, Institut Pasteur, INSERM, F-75015 Paris, France; 4 Sorbonne Universités, Université Pierre et Marie Curie, Complexité du Vivant, F-75015 Paris, France; 5 CNRS, Paris, France; 6 Paris-Saclay Institute of Neuroscience (Neuro-PSI), UMR 9197, Université Paris-Saclay, CNRS, F-91405 France

**Keywords:** Neural circuits, Sensory processing, Long-term memory

## Abstract

Multisensory interactions are essential to make sense of the environment by transforming the mosaic of sensory inputs received by the organism into a unified perception. Brain rhythms allow coherent processing within areas or between distant brain regions and could thus be instrumental in functionally connecting remote brain areas in the context of multisensory interactions. Still, odor and sound processing relate to two sensory systems with specific anatomofunctional characteristics. How does the brain handle their association? Rats were challenged to discriminate between unisensory stimulation (odor or sound) and the multisensory combination of both. During learning, we observed a progressive establishment of high power beta oscillations (15–35 Hz) spanning on the olfactory bulb, the piriform cortex and the perirhinal cortex, but not the primary auditory cortex. In the piriform cortex, beta oscillations power was higher in the multisensory condition compared to the presentation of the odor alone. Furthermore, in the olfactory structures, the sound alone was able to elicit a beta oscillatory response. These findings emphasize the functional differences between olfactory and auditory cortices and reveal that beta oscillations contribute to the memory formation of the multisensory association.

## Introduction

Does this apple sound good? The smell and sound produced by biting an apple are likely to influence each other^1^. While vision and audition, dominant in human perception, typically prevail among cross modal interaction studies, other modalities (e.g. smell, touch^2^) may be as efficient in modulating or generating multisensory internal representations. Integration of information from different sensory systems is inherent to unified perception but how the brain combines them is still poorly understood. The traditional feedforward view of multisensory interactions processed exclusively in associative cortices has recently been challenged by data showing integration occurring at early stages of perceptual processing^3^. Such studies rather envision multisensory integration as the combination of multiple processing within distributed networks than the specific task of associative cortices gathering convergent information from unimodal cortices^4,5^. Integration could be performed directly through low-level multisensory processing in primary sensory areas^6^ or indirectly through preparatory states and selective attention^7,8^.

Because synchronous oscillations are believed to support local and large-scale integration of brain processes^9^, they may also provide a mechanism for cross-modal integration^10–13^. Indeed, an increase of oscillatory power has been observed in humans during multisensory processing^10,14,15^. During visuo-tactile stimulation in rats, the visual stimulus induced a reset of ongoing neuronal oscillations and enhanced the power of oscillations induced by the tactile stimulation in the somatosensory cortex^16^. In the context of maternal behavior in mice, exposure to pups’ odor can modulate neuronal responses to pure tone and natural auditory sounds in the primary auditory cortex of the mother. This olfactory-auditory integration appeared in lactating mothers and also in naive virgins that had experience with the pups, showing that these long term changes are likely to be experience dependent^17,18^. Could neuronal oscillations subserve communication between distant brain areas, spanning sensory and associative regions during multimodal associations?

Here, by recording LFPs, we examine the contribution of neuronal oscillations for a multimodal association between a sound and an odor in the context of operant conditioning discrimination in freely behaving adult rats. In rodents, oscillations related to odor coding and memory are prominent in the olfactory system^19^ while their presence is more controversial in the auditory system where most of the studies have focused on spike timing temporal precision^20,21^. Our objectives were to challenge two primary sensory areas, having very different oscillatory behaviors toward sensory processing, to bind together in the context of a multisensory task; and to examine multimodal processing in sensory vs. integrative brain regions.

To this aim, we sampled a network of structures including primary sensory areas -olfactory bulb (OB) and primary auditory cortex (A1)- and heteromodal areas -piriform cortex (PC) and perirhinal cortex (Prh)- simultaneously. An increasing number of studies in rodents has identified the piriform cortex (or olfactory cortex) as a multisensory integrative center influenced by gustatory, auditory and tactile inputs^22–24^. Direct connection between A1 and the PC have been identified in gerbils^25^. The Prh, on the other hand, primarily receives inputs from the auditory and olfactory areas, and could participate in cross modal binding^26,27^. In this study, a signature of multisensory activity was observed; higher beta oscillations (15–35 Hz) observed in the Local Field Potential (LFP) recordings were elicited by the odor-sound association compared to the odor alone in the PC and the OB. Interestingly, A1 seemed reluctant to such associative learning. We also provide evidence that in the context of our task, plasticity in primary sensory areas was influenced by multisensory processing. Indeed, at the end of the learning process, the olfactory regions OB and the PC took part in the auditory stimulus processing.

## Results

### Rats can learn to associate an odor and a sound

We designed a multisensory task in which rats were trained to exhibit a differential behavior in response to unisensory stimulation (odor or sound alone) versus the multisensory combination of both odor and sound. 8 rats were engaged in the multisensory test, the other being excluded because at least one electrode was not at the right localization, or had bad signal, or because the animal was not able to perform the habituation.

**Figure 1:**
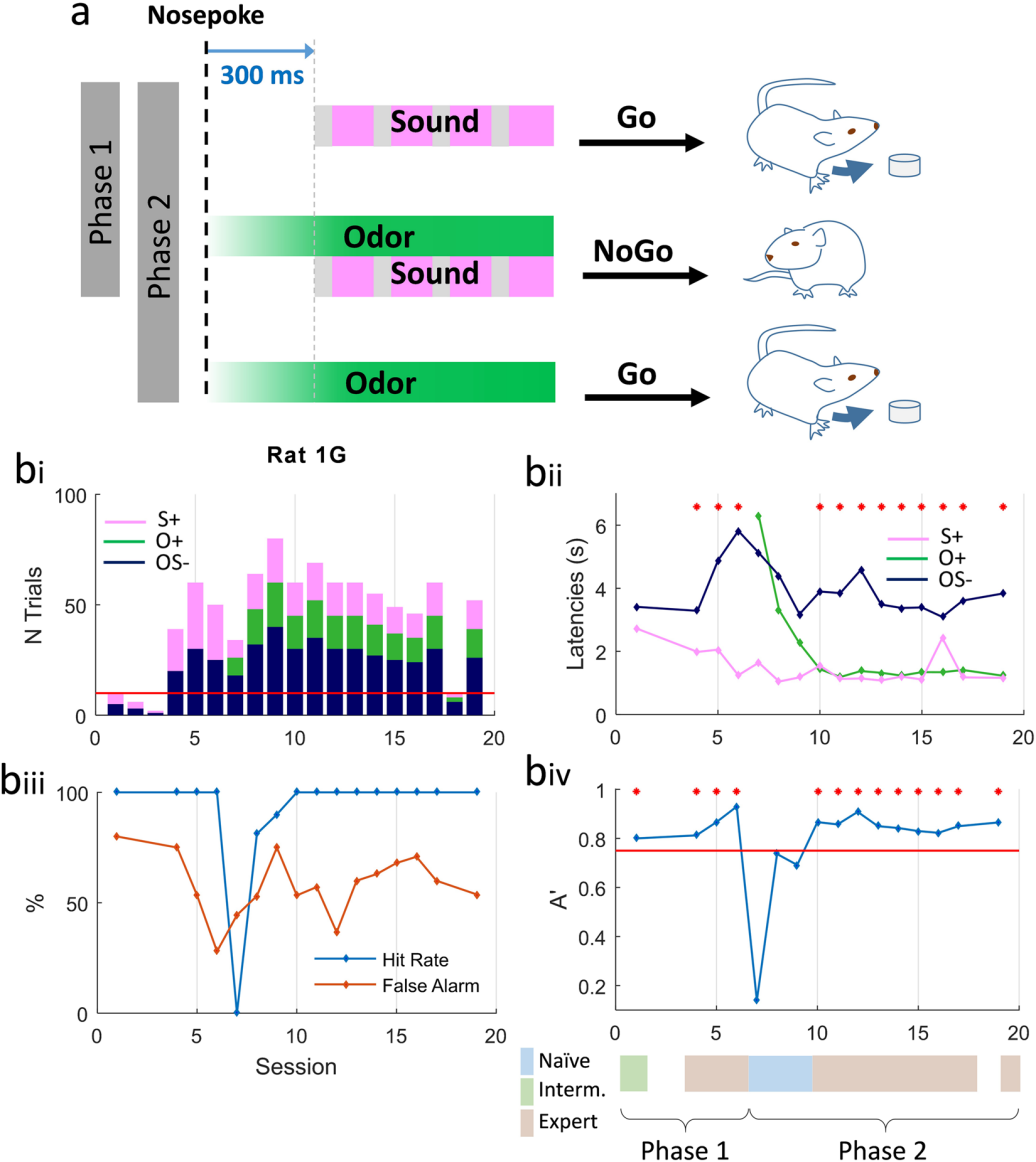
Task design and behavioral performances. (**a**) Multisensory task design. During Phase 1, two stimuli were randomly presented, sound (S+), rewarded by a sucrose pellet and odor/sound (OS−) not rewarded. In Phase 2, the third stimulus, odor (O+) rewarded by a sucrose pellet was added. Stimuli were triggered by a nosepoke, the odorant delivery started immediately, the sound, a sequence of four 50 ms white noise bursts separated by 200 ms, started 300 ms after the nosepoke. Rats received 50% of OS− stimulations, 25% of O+ and 25% of S+ so that in one session, 50% of the trials were rewarded. **(b)** Example of behavioral parameters for one representative rat. (**bi**) Number of trials of each stimulus performed by the animal during each session. (**bii**) Example of the evolution of latencies corresponding to each stimulus during learning. Latency (in s) was measured for every trial as the duration between nose poke in the stimulus port and nose poke in the reward magazine (correct No-Go was assigned a maximum latency of 6.3 s after the nose poke onset). Each value represented the average latency in the session for one stimulation. O+ was introduced at session 7 for this animal. Red dots indicate sessions for which OS− latencies were significantly different from O+ and S+ latencies (Student t-test p < 0.05). Missing points correspond to excluded sessions with less than 5 trials. (**biii)** Percentage of hit rate (Go trials, blue line) and false alarms (NoGo trials, red line) for all the sessions. (**biv**) A′ index calculated for each session. Colorbar below the graphic indicates the level of the animal according to the two index t-test and A′. Threshold for discrimination was set up at 0.75 (red line and red dots).

The task (Fig. 1a) was divided in two phases. During **phase 1**, the animal learned that the sound stimulation (S+) was associated with the delivery of a food pellet, and that the odor/sound stimulation (OS−) was not rewarded. Figure 1bi–iv exemplifies performances for one rat. The latencies for S+ and OS− progressively diverged toward a quick move to the food tray (Go response) in response to S+ and a motor inhibition (NoGo response) after OS− sampling (Fig. 1bii). 8 rats reached the criterion set at 70% correct responses with an average (±SD) of 4 ± 3 daily sessions.

In **Phase 2**, rats had to discriminate between 3 stimulations: sound (S+; Go), odor (O+; Go) and odor/sound association (OS−; NoGo). During the first session of Phase 2, latencies were high for the newly introduced odor stimulation (O+). In the following sessions, as the rat associated the odor delivery with the possibility of obtaining a reward, the latency after O+ progressively shortened. It reaches similar values than for S+ at the end of the task, resulting in a different behavior by the animal for the unisensory and multisensory stimulations. Acquisition of Phase 2 took longer with an average (±SD) of daily 17 ± 3 sessions to reach the criterion set at 70% correct responses. 5 rats reached the criterion.

Because the time course of the acquisition of the task varied across animals, we used two indices to compare between similar levels of learning: i) a student t-test to compare average latencies in response to OS− stimulation vs. O+ and S+ stimulations; ii) an A′ sensitivity index (see methods) to discriminate between good hits and false alarms (Fig. 1biii,biv). Based on these indices, we sorted each session of the task as naïve (p ≥ 0.05 and A′ ≤ 0.75), intermediate (either p ≤ 0.05 or A′ ≥ 0.75) and expert (p ≤ 0.05 and A′ ≥ 0.75) (Fig. 1biv, bottom). Group results for behavioral performance are shown in Supplementary Fig. 1. Note that rats also learn the task if the unisensory stimulus was odor instead of sound in phase 1 (Suppl. Fig. 2).

### Beta oscillations increased during odor-sound multimodal learning

**Figure 2:**
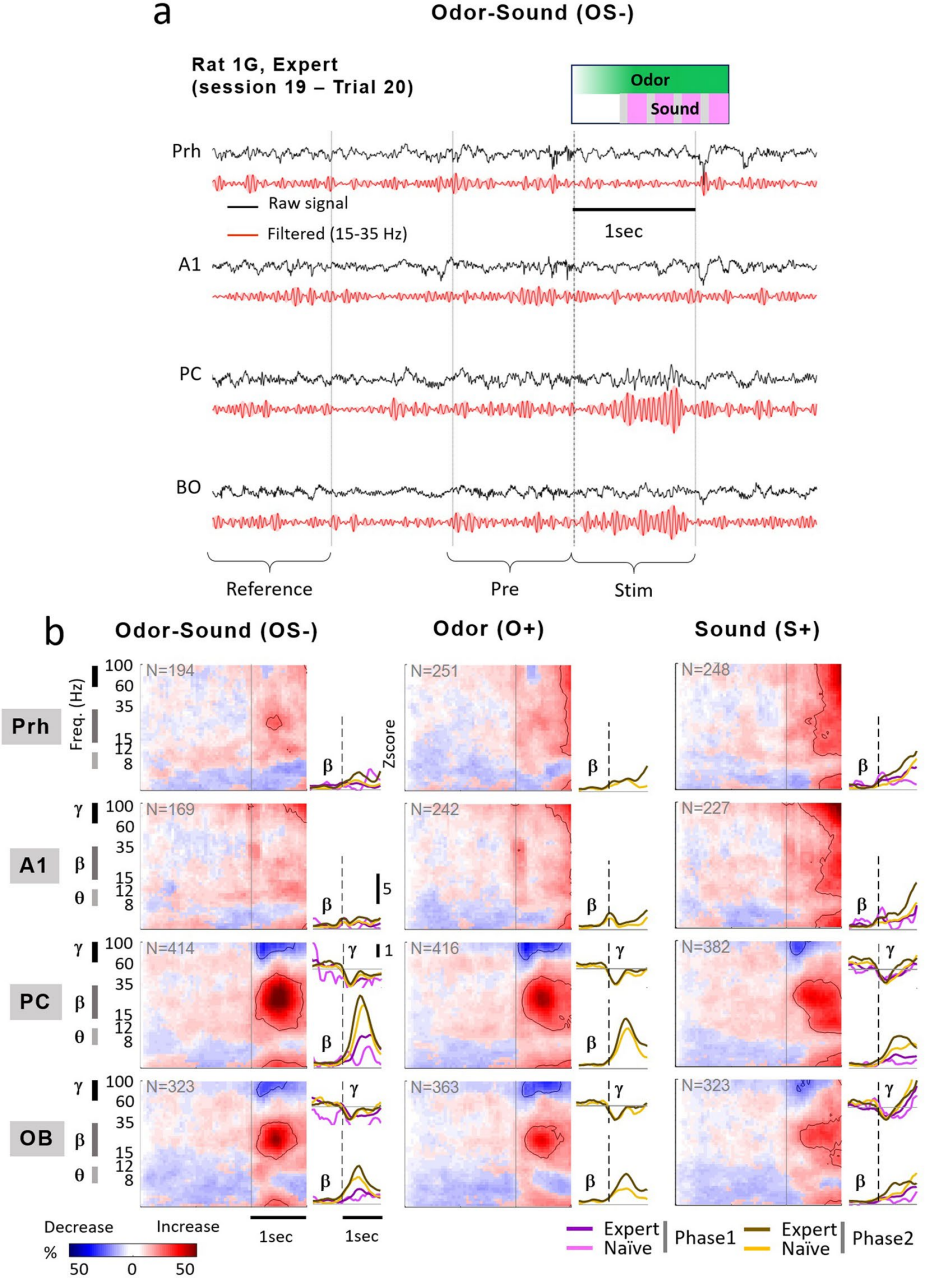
Evolution of oscillatory power during learning for the uni- and multisensory stimuli. (**a**) Raw and filtered (15–35 Hz) representative LFP traces from the OB, PC, A1, Prh during a single trial from a session from Phase 2 expert. Stimulation was OS−. Indicated below are the time windows used for the analysis: reference ([−3 −2] s relative to the nose poke in the stimulus port), Pre ([−1 0] s) and Post ([0 1] s). **(b)** Time-frequency maps of oscillatory activity during Phase 2 expert for each brain areas (lines) displayed for the 3 stimuli: OS− (left), O+ (middle) and S+ (right). Wavelet analysis has been performed between 0 and 100 Hz. Each plot reports the percentage of trials showing a significant increase (red) or decrease (blue) of LFP power at each time relative to the reference period ([−3 −2] s) as a function of time (x-axis) and frequency (y-axis, log scale). Solid lines are drawn around regions showing a modification for more than 25% of the trials. Major effects were observed in beta (15–35 Hz) and gamma (60–100 Hz) frequency bands. Inserted yellow and purple lines represent the corresponding profile of z-score normalized power in the beta band as a function of time, from −0.5 s to 1 s relative to the nose poke (dotted vertical line). For the 3 stimuli, power for each learning level (naïve and expert, Phase 1 and 2) was represented by different color lines.

LFP signals were recorded in the OB, A1, anterior PC and Prh while rats performed the multisensory task. We examined changes in LFPs associated with stimuli discrimination learning in naïve, intermediate, and expert levels (Phase 1 and 2). As described previously, the task was not easy for the animals, with a performance threshold set at 70% equating success. Next, to ensure we analyzed a condition in which rats are performing multisensory integration, we selected only the trials for which rats gave a correct response (good hit or correct rejections) from Phase2 experts. Analyses of the LFP were centered on the stimulation period (Fig. 2a,b).

In the OB and PC, when the animals responded correctly to multisensory (OS−) and unisensory (O+ and S+) stimuli, all stimuli elicited a significant reduction of power in the gamma band (60–100 Hz) concurrent with an increase in the beta band (15–35 Hz) in 25% to 60% of trials as revealed by time frequency analysis (see contours in Fig. 2b). Beta increase was more frequent in the PC than in the OB.

Changes in gamma and beta power due to learning of Phase 1 and Phase 2 are illustrated in the power profiles of Z-score averages for the different levels (Fig. 2b, insets on the right). Beta oscillations, but not gamma, built up during learning in PC and OB (ANOVA 3 ways - stimulus, area, learning phase- for beta oscillations; significant effects according to the learning level (df = 1,12109), brain area (df = 3,12109) or in interactions area*stimulus (df = 6,12109) and area*learning phase (df = 9,12109): all p < 10^−10^). Beta power associated with the multisensory stimulation (OS−) progressively increased in the PC and in the OB during Phase 1 (post-hoc t-test, naïve vs expert Ph1, all p < 2.10^−3^, p > 0.13 in Prh and A1) and in PC, OB and Prh during Phase 2 (post-hoc t-test, expert Ph1 vs expert Ph2, all p < 3.10^−3^). A strong beta power was already associated to O+ in the olfactory structures when introduced in Phase 2 naïve level because the rats had already learned this odor in the stimulus OS−. S+ stimulus induced an increase in beta power in OB and PC compared to the reference period in Phase 1 expert (t-test, all p < 10^−9^). This activity gradually expanded during Phase 2 (OB, PC, Prh, naivePh2 vs expertPh2, post-hoc t-test, all p < 4.10^−5^). Then, once associated to the odor by learning, the sound alone (S+) also elicited beta oscillations in the OB and PC. These oscillations were induced and different from the evoked response to the temporal envelope of the sound alone (S+) observed only in A1 (Suppl. Fig. 3). On the other hand, no auditory oscillatory responses could be observed in A1 during the task.

**Figure 3:**
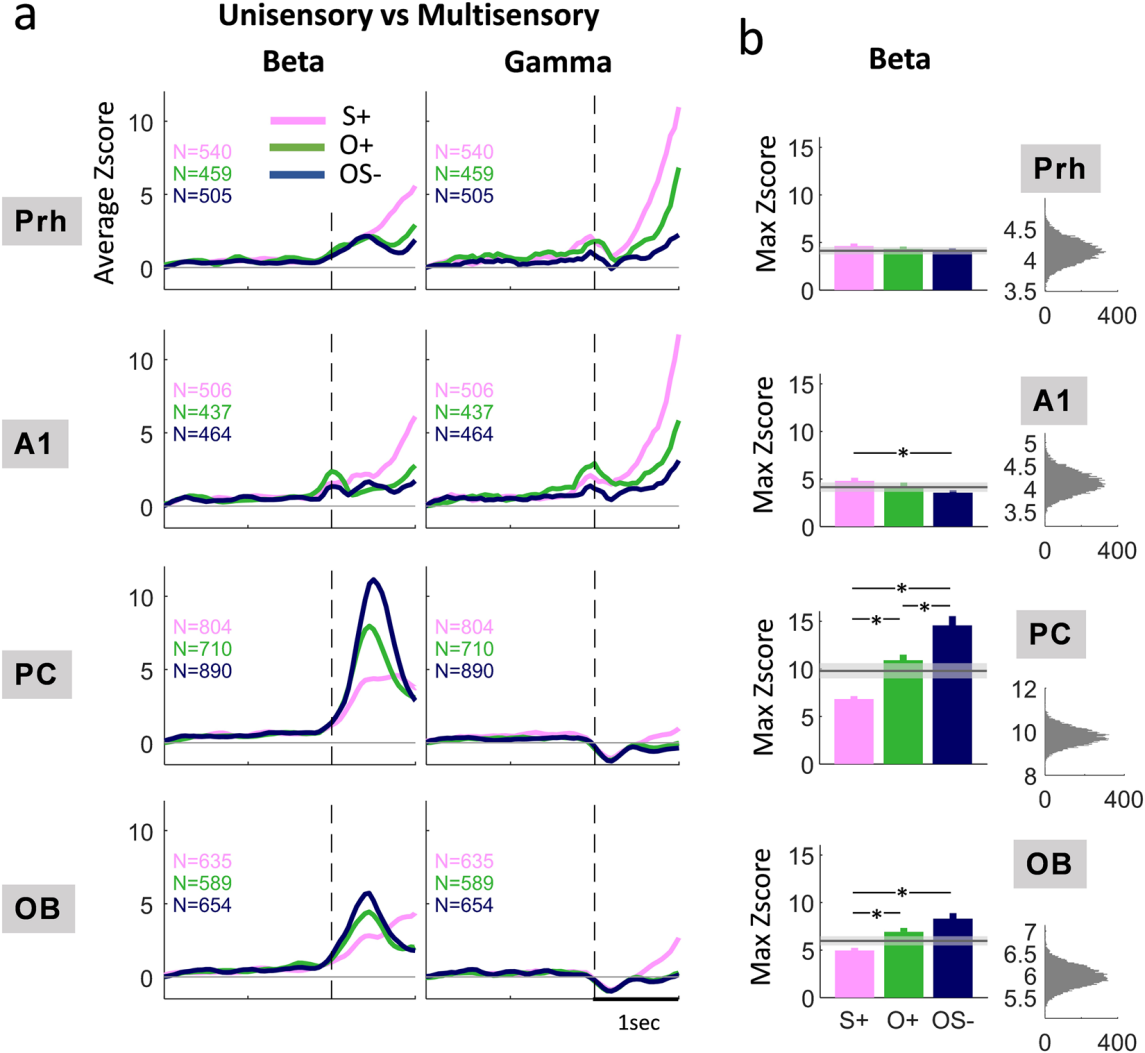
Power of oscillations in beta (15–35 Hz) and gamma (60–100 Hz) bands in uni- and multimodal conditions. (**a**) Profile of z-score normalized power in beta (15–35 Hz) and gamma (60–100 Hz) bands as a function of time, from –2 s to 1 s relative to the nose poke (dotted vertical line). Power is represented for each stimulus as a different color line. Only success trials of Phase 2 are selected. The number of trials is indicated for each condition. **(b)** Left: maximum averaged Zscore in the beta band during stimulus sampling (calculated in the time range [0.3 0.6] s). Data are displayed as mean +/−s.e.m. Same color code as in a) *p < 0.05/3 (Bonferroni on testing pairs). The gray line is the average of Z-Score computed from a linear summation of O+ and S+ trials (see methods). The shaded gray shows the 2 SD interval around the mean. Right: Distribution of the 10000 bootstrapped samples of average Z-Score computed from the linear summation of as many O+ and S+ trials as OS− trials.

Z-Score values corresponding to each stimulus were compared during the first second after the nose poke (Fig. 3). In the PC, beta oscillations power was significantly enhanced for the multimodal association between odor and sound (OS−) compared to the odor or the sound presented alone (O+ and S+) (ANOVA 1 way - stimulus, df = (2,2537), p < e-10; post-hoc t-test, S+ vs OS−: p < e-10, O+ vs OS−: p = 4e-4). This increase occurs for most animals (Suppl. Fig. 5). The response to the multimodal stimulation did not exceed the arithmetic sum of unisensory responses. However, we used a bootstrapping method to study the distribution of Z-Score values that would be obtained from the linear summation of S+ and O+ trials (Fig. 3b). We observed that not only beta oscillations power for S+ and O+ combinations was way below that for the multimodal association but it was also weaker than for the odor alone. This result suggests that beta oscillations elicited by S+ are not coherent with those elicited by O+ and that the multimodal association really enhances beta power in a superadditive way. In the OB, beta power was higher for OS− than for S+ (ANOVA 1 way - stimulus, df = (2,2740), p = 5.4e-6; post-hoc t-test, S+ vs OS−: p = 1.4e-4) but not significantly different between O+ and OS− after Bonferroni correction (post-hoc t-test, O+ vs OS−: p = 3.6e-2 > 0.05/3). However, as previously, the use of the bootstrap procedure suggested a superadditive enhancement of beta oscillations power compared to unisensory modulations. Remarkably, no multimodal effect could be observed through beta oscillations in the other associative area Prh: OS−, O+ and S+ elicited a weak beta power increase (see Fig. 2b and Z-Scores between 3 and 5 in Fig. 3a), with no difference between them (ANOVA 1 way – stimulus, df = (2,2215), p = 0.07), and no change in the gamma band. Noteworthy, we did not observe a reset of the phase of beta oscillations by the sound in the olfactory structures (Suppl. Fig. 4). Analysis of the Phase Concentration Index (PCI) across trials showed strips spreading over beta and gamma bands at each noise burst in A1. These are very short duration and stem from the phase-locking of the high-frequency auditory evoked potential to the auditory stimulus (noise burst), but they do not correspond to oscillations. Interestingly, strips are sharper for the OS− condition compared to the S+ condition, showing a better reproducibility across trials of the auditory evoked potentials, even if the shape and amplitude of auditory evoked potentials are similar between those two conditions (Suppl. Fig. 3).

To sum up, once the rats solve the multimodal association, beta oscillations power increased for all the stimuli, including S+ in the OB and PC, and in Prh for O+ and OS−. We did not observe phase reset in our paradigm, but the multimodal stimulus OS− induced a stronger beta power with a superadditive effect relative to unimodal stimuli.

### Multisensory association changes the strength of feedback input of PC onto OB

**Figure 4:**
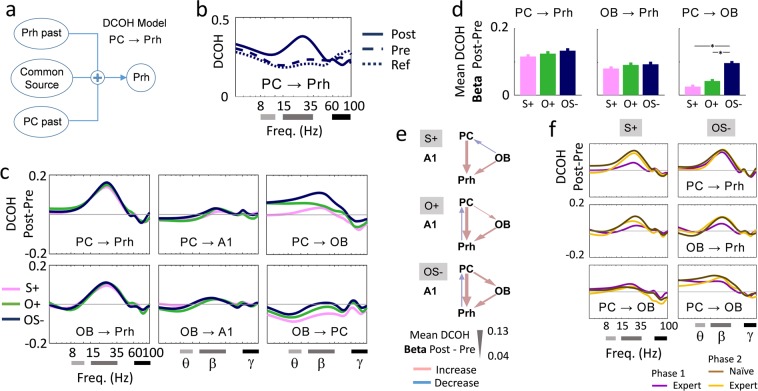
Averaged directional coherence (DCOH) in uni- and multimodal conditions. (**a**) DCOH model applied to the coupling from PC to Prh **(b)** Raw DCOH value for the coupling from PC to Prh and for each time range Ref [−3 −2] s, Pre [−1 0] s and Post ([0 1] s. the DCOH index (y-axis) is represented as a function of the frequency (x-axis, log scale). Gray lines represent the Theta, Beta and Gamma band of interest. **(c)** For the 3 stimuli represented as different color lines, flows between pairs of structures were estimated as the difference of DCOH between the stimulation period ([0 1] s) and the Pre period ([−1 0] s). Only success trials of Phase 2 are selected. **(d)** DCOH Post-Pre in the beta band (15–35 Hz). Data are displayed as mean +/−s.e.m. *p < 0.05/3. **(e)** Summary for the beta band of the significant changes in directional flow (coupling, pink arrows; uncoupling, blue arrows) between PC, OB and Prh for all rats. Mean DCOH Post-Pre values are illustrated by the arrows thickness. **(f)** Effect of learning on DCOH Post-Pre. Only success trials are selected.

We next examined information transfer within the structures recorded by using directional coherence (DCOH estimate), a method based on the temporal relation between two or more signals in the frequency domain. DCOH analysis was conducted on all the sessions performed by the rats for the three stimulations. The largest changes elicited by stimulus sampling were observed in the beta (15–35 Hz) frequency band (Fig. 4).

We focused the analysis on the DCOH estimate between the stimulus period (Post, [0 1] s) and the Pre period ([−1 0] s) in the beta (15–35 Hz) frequency band (Fig. 4b). For the success trials of Phase 2, a large increase of DCOH_Post-Pre_ was observed from the OB to the Prh and from the PC to the Prh with no difference between the 3 stimuli (difference between O+, S+ and OS−; ANOVA 1 way – stimulus: OB− > Prh, df = (2,1275), p = 0.40; PC- > Prh, df = (2,1409), p = 0.22). DCOH_Post-Pre_ differed between the stimuli from PC to OB (ANOVA 1 way – stimulus, df = (2,1762), p < e-10). Indeed, DCOH_Post-Pre_ was significantly higher in response to OS− than in response to O+ and S+ (Fig. 4c,d; post-hoc t-test, S+ vs OS−; O+ vs OS−: p < 1e-9). O+ did not generate a stronger coupling from PC to Prh than S+.(Fig. 4c,d; post-hoc t-test, O+vs S+: p = 0.05).

As for the power of oscillations in the different structures, DCOH was enhanced throughout the different learning steps (Fig. 4f); in particular, increase in DCOH coupling from PC to Prh was delayed for S+ (ANOVA 1 way – learning level, df = (2,680), p = 1e-8; post-hoc t-test, expert Ph1 vs naïve Ph2, p = 3e-6) compared to OS− (ANOVA 1 way – learning level, df = (2,539), p = 0.59).

### Frequency band related activity was little predictive of the behavioral output

**Figure 5:**
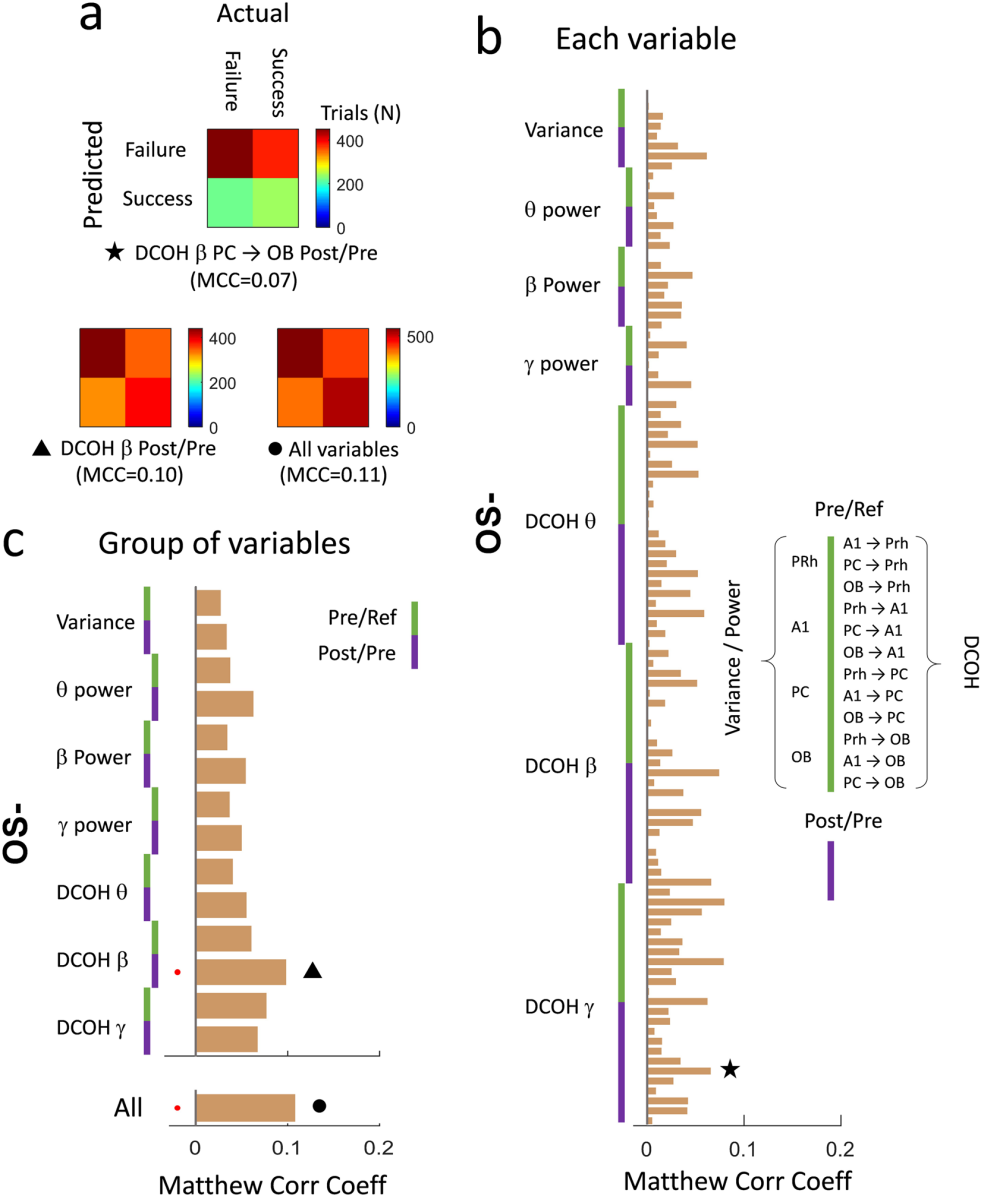
Prediction of behavioral output using frequency band related activity. (**a**) Individual example of confusion matrices: for a given variable (top matrix: DCOH Post-Pre averaged over the beta frequency band for the flow from PC to OB), each available trial is assigned to the closest average of failure or success trials. The Matthew Correlation Coefficient (MCC) is a measure between 0 and 1 quantifying how diagonal is the matrix and therefore how well separated is the population of failure and success trials. MCC values therefore represent the ability of a variable to correctly predict the behavioral output (Go or No Go) for a given trial. This analysis can be done for the group of variables DCOH Post-Pre Beta between all possible pairs of brain areas (bottom left matrix) or all available variables (bottom right matrix). **(b)** MCC associated with the difference between Post and Pre (green set) or Pre and Ref (Purple set) of time signal variance, power or directional coherence related to frequency band activity. **(c)** Same analysis than in (**a**) while grouping variables from all brain areas as one new variable (top plot) or grouping all variables together (bottom plot) as in (**a**).

Oscillations in the beta frequency band are correlated with learning and multisensory processing in the olfactory areas: amplitude and coupling between areas progressively increased during training and were stronger for multisensory than for unisensory stimulations. Thus, we examined whether beta band activity within the sampled network of olfactory and auditory areas would carry enough information to predict the behavioral output of the animal. We therefore examined to what extent the time signal variance, the power or directional coherence related to frequency band activity, could be predictive of success or failure in the context of OS− stimulation trials (Fig. 5). In brief, we built up confusion matrices displaying the value of a given variable or a group of variables to the closest average value between failure or success trials (see methods and Fig. 5c). We observed that overall, the network activity that we studied has little or no predictive power on the behavior output of the animal. DCOH Post-Pre in the beta band (15–35 Hz) was the most reliable variable to predict failure or success of a trial (Fig. 5a,b) but performance remains weak (10% of variance explained).

## Discussion

We challenged rats on a task where they had to distinguish between the association odor/sound and an odorant alone or a sound alone. The analysis of LFPs recorded during the acquisition of the task revealed the establishment, during learning, of high power beta oscillations (15–35 Hz) spanning the olfactory bulb, the piriform cortex and the perirhinal cortex, but not the primary auditory cortex. In the PC of expert animals, these oscillations, through the analysis of power and directed coherence, were found to be distinct between unimodal stimuli (odor or sound) or the multimodal association of them (odor + sound). Beta power elicited by the multimodal stimulus was higher with a superadditive effect (with regards to the bootstrap method used here). Furthermore, the directional coupling from the PC to the OB was stronger for the multimodal compared to unimodal stimuli. These variations of beta oscillations at the level of the PC provide evidence for a role of this structure in multimodal processing. We assume that beta oscillations convey information about the sensory properties of the stimulus and its significance for the animal; however, their characteristics (power, coherence etc…) are not powerful predictors of the behavioral output.

If beta oscillations helped identify regions of the brain involved in the task, why did not we see beta oscillations in A1? As described above, oscillations are prominent in olfactory structures. There is no consensus on the presence or lack of relevant oscillations in A1 in rodents, especially in awake conditions^28^. Long lasting gamma oscillations (40–140 Hz) recorded in A1 have been proposed to be predictive of learning acquisition in the context of fear conditioning^29,30^. Evoked gamma-band activity following passive or active auditory stimulation has also been observed in cats or gerbils^31,32^. In our data, the significant activity observed in the gamma band in A1 (Fig. 2b) was late and likely due to animal movements or noise artifacts since we did not observe a similar activity during OS− stimulation, even if we cannot exclude that auditory evoked gamma band activity would be down-modulated by the presence of the odor.

Lakatos and colleagues (2007) examined somatosensory-auditory interactions in the macaque monkey. They found that unimodal somatosensory stimulation induced phase-locked oscillations in A1 due to a reset in phase of ongoing oscillations in the structure. They thus propose a potential mechanism for multisensory interactions^33,34^: the input of one sensory modality could reset the phase of the oscillations in a cortex processing another modality, leading to a better sensory integration. This effect has also been observed for visuo-tactile associations^16^. Given that odorants are volatile and arrive gradually in the nose, shaped by respiration, we did not expect an odorant driven phase reset in A1. We also did not observe a reset of the phase of beta oscillations by the sound in the olfactory structures. We cannot exclude that in our data, the way our stimuli are presented masks phase resetting, as it has recently been shown that phase synchrony occurs in an olfactory-auditory integration in human when the two modality did not arrive simultaneously^35^. However, using PCI analysis we detect sharper strips for the OS− condition compared to the S+ condition (Suppl. Fig. 4). This effect is difficult to interpret insofar as the shape and amplitude of auditory evoked potentials are similar between those two conditions (Suppl. Fig. 3). A better attentional drive due to the presence of the odor in the OS− condition may actually contribute to these higher PCI values by reducing EEG noise for instance, compared to the S+ condition.”

Since the phase resetting cannot explain the increase in beta oscillations in PC, we suggest that it relies on plasticity within the olfactory network. Beta oscillations are often considered as the ‘top-down’ rhythm in opposition to ‘bottom-up’ gamma oscillations^36–39^. In olfactory areas, beta oscillations have been extensively studied during memory tasks and correlated with olfactory recognition in a broad network of brain areas, including limbic and olfactory areas^40–47^. The establishment of a network through beta oscillations during the multisensory task was similar to what had been previously found in the aforementioned studies concerning unisensory odorant discrimination. In addition, a buildup of oscillatory power in the same frequency range has been observed in the anterior PC for olfacto-tactile tasks^48^.

Beta power increase together with enhanced PC to OB directional coupling suggested that top-down modulation exerted onto the OB was stronger in the multisensory condition. Indeed, the odor association learning depends on feedback projections^49^ and triggers beta oscillations enhancement in both the PC and OB^40^. In the context of unimodal olfactory learning, this enhancement was abolished when the PC to OB connections were disrupted^41^.

What triggered this increase in feedback signal onto olfactory areas? We hypothesized that the signal developed as the animal built the representation of the multisensory object.

Nevertheless, we cannot exclude the involvement of other neuronal processes. For instance, it is possible that beta oscillations were enhanced for the multimodal stimulus because its processing required a stronger attentional drive^37^. Also, the multisensory task that we developed was asymmetrical since the three stimuli did not have the same behavioral output (Go or NoGo) and the multisensory stimulus is always a Go response, which could have influenced the power of beta activity elicited by the stimulus. For instance, beta oscillations could be modulated by motor planning^50^ or reward expectation. However, in previous studies using only olfactory cues, the NoGo stimulus was not specifically associated with a stronger beta power than the Go stimulus^40,51,52^. Additionally, O+ and S+, associated with the same reward, had distinct oscillatory signatures in the olfactory regions, pleading for the fact that beta oscillation power was not strictly related to the expectancy but also reflected sensory processing.

Moreover, strength and flows of beta activity to Prh were of comparable levels in failed and successful trials, which have opposite motor output (Suppl. Fig. 6). We cannot exclude that NoGo condition favors (or, equivalently, Go condition mitigates) the beta flow from PC to OB in the presence of odors (Suppl. Fig. 6b). A possibility to make the task more symmetrical might be to use tests based on n-alternative choice. Since alternative choice based tests already involve longer response latencies than Go/NoGo in olfactory tasks^53^, that rats are able to learn a multimodal alternative choice task remains to be shown. Indeed, as seen with performances of the rats, our task was already difficult to learn, probably because it involved negative patterning: the presentation of either of two stimuli alone was reinforced, but conjoint presentation was not: A+, B+, AB−^54^.

Parahippocampal areas such as the Prh, recorded in the present study, are thought to be required for the building of a configural representation. Beta increase was modest in the Prh during stimuli sampling, and identical for the three stimuli. Interestingly, we found that PC and OB were always driving Prh at the beta frequency band, even for the sound stimulation. We focused our study on sensory areas but other brain regions not examined in this study have also been shown to be involved in multisensory processing such as the lateral entorhinal cortex or the hippocampus^2^, where beta oscillations had also been recorded during learning^43,44,48,52^.

The auditory cortex can be modified by olfactory inputs as shown by Cohen *et al*.^18^ who found at the level of single cells, that the odor of the pup was able to change the way A1 encoded the sound emitted by the pup, and that this effect builds through learning^17^. However, it is possible that plastic abilities of A1, albeit present, do not extend to substantial modifications of their neural code even following behavioral conditioning, which would be detrimental to neural stability. In other words, we suggest that, because of the kind of stimulus that it processes, or because of its structure, the network of A1 may not give rise to sustained beta oscillations such as those observed in olfactory structures. Other mechanisms should mediate the influence of the odor in A1. Conversely, A1 could still be involved in the multimodal network at stake here using neural activities other than oscillations and blind to the LFP that we recorded.

On the contrary, in olfactory structures, data showed that once the odor and the sound have been associated by learning, even the sound alone was able to elicit a beta oscillatory response in the anterior PC and to a lesser extent in the OB. Then, the olfactory structures can learn to “smell sounds”. Indeed, we know from other studies that the PC is responsive to auditory inputs. Varga and Wesson^23^ described that 29% of units sampled within the PC of mice display tone-evoked responses. However, the same team did not find auditory responsive neurons in the OB^55^. This finding reinforced the view of the PC as a multimodal associative area, which can be modulated by auditory, visual^56^, gustatory^22^ and tactile sensory inputs^24^. The PC is also a key structure for odor-related associative memory as it has been shown by multiple studies^43,57–60^. Indeed, in our task, the PC was the first brain region, among those we recorded, that presented an enhancement of beta band power with learning.

These results are also consistent with what has been reported for audio-visual stimuli learning^61^ or odor-images association in humans^56,62,63^. In people trained in a visual olfactory associative learning, fMRI data showed that the conditioned visual cue elicited activity in the PC in the absence of odor^56^. This was also true in an episodic memory task, after the explicit encoding of odor/images associations. In this case, the presentation of a visual cue was sufficient to trigger an activity specific of the related olfactory content in the PC. The authors interpret that activity as being an incidental reactivation of the different part of the memory content^62^. We can similarly interpret our findings and compare them to the concept of “redintegration” taken from the psychology whereby a part reinstates a previous whole^64,65^. The recall of only one of the components of the multimodal pair was enough to cause an activation of the entire network. This associative learning suggests the formation of a multimodal internal representation. Memory related beta oscillations could be instrumental in establishing meaningful cross-modal integrations.

## Methods

### Subject and surgery

Experiments were carried out in accordance with the European guidelines regarding care and use of animals for experimental procedures. All studies were conducted on groups of Wistar rats (250–300 g) purchased from Charles River Laboratories. All the procedures have been approved by the ethical committee N°059 and the project is referenced by the French Ministry of Research as APAFIS#1 130-2015071009549877.

15 rats were implanted with electrodes in the olfactory bulb (OB), the piriform cortex (PC), the perirhinal cortex (Prh) and the primary auditory cortex (A1). For the surgery, rats were anaesthetized with equithesin (a mixture of chloral hydrate and sodium pentobarbital, 3 ml/kg, i.p.) complemented by i.p. injection of buprenorphine and a local subcutaneous injection of lidocaine. Two wires recording electrodes (125 µm diameter stainless steel polyimide coated wires; PlasticOne, USA) were implanted unilaterally in the OB (antero-posterior (AP) 8.5 mm relative to bregma, medio-lateral (ML) 1.5 mm and 3 mm in depth). Monopolar electrodes were implanted unilaterally in the anterior part of the PC (AP 2.2 mm, ML 4 mm, 7,4 mm in depth), the Prh (AP - 3 mm, ML 4 mm with an angle of 14°, and 7,4 mm in depth) and A1 (AP -5 mm, ML 4 mm along the temporal bone), see Supplementary Fig. 7. Electrodes depth was adjusted individually. In the OB, electrodes were lowered to the mitral cell layer using electrophysiological monitoring. In the PC, monopolar electrode was lowered near the pyramidal cell layer using the evoked potential induced in response to short electrical stimulations of the OB electrodes (0.1 ms pulse, 300 µA, Suppl. Fig. 7). In A1, electrode location was set using sound-evoked field potential in response white noise (Suppl. Fig. 7).

The reference electrode was positioned in the skull bone above the contralateral cortical hemisphere at 5 mm posterior to the bregma. All electrodes were connected to a miniature socket (PlasticOne, USA) fixed onto the rat’s skull by dental cement after the application of a surface activator (Super-Bond C&B, Sun Medical). Two weeks of recovery separated surgery from recordings.

After experiments were complete, rats were given an overdose of Pentobarbital. Brains were extracted for histological examination (Suppl. Fig. 7).

### Behavior

Experiments were performed in 50 × 25 × 30 cm size arena (Habitest, Coulbourn, USA) controlled by a computer (GraphicState software, Coulbourn USA). One wall of the cage was equipped with a stimulus port, the opposite wall was equipped with a reward magazine in which the animal could receive 20 mg precision sugar pellets (BioServ, USA). A speaker was located behind the stimulus port. Photobeam sensors mounted on the sides of the stimulus port and the reward magazine monitored nose pokes. Deodorized air constantly flowed through the stimulus port (1.9 L/min). Detection of nose pokes in the stimulus port was used to trigger sensory stimulations.

The olfactory stimulation was delivered using an automated olfactometer (ValveBank II, AutoMate Scientific) that controlled the duration and the flow rate of the stimulation. A piece of filter paper, loaded with 50 µL of an odorant solution (3% of pure hexanal (Fisher Scientific UK) diluted in mineral oil) was used to odorize the airflow. Odorant stimulation lasted for 1.3 s.

The auditory stimulation was a sequence of four 50 ms white noise bursts separated by 200 ms (onset to onset) and presented at 75 dB SPL. The sequence started 300 ms after the nose poke in the stimulus port.

The multisensory stimulation was the combination of both stimuli: the odor triggered by the nose poke in the stimulus port plus the sound that started 300 ms later. This delay was set according to the calculated time spent by the odorized airflow to travel from the valve to the stimulus port in the device (270 ms with a 1.9 L/min airflow). This ensured that the rat simultaneously perceived both the odor and the sound.

During habituation (2–3 days), food-restricted animals were trained to associate a nose poke in the stimulus port (no stimulus) with the release of the sucrose pellet in the reward magazine. The test was divided in two phases.

#### Phase 1: Sound (S+) vs. Odor/Sound (OS−) discrimination

Stimulus onset was initiated by the rat nose poke in the stimulus port. Two stimuli were randomly delivered: the sound (S+, 50% of the trials) and the association odor/sound (OS−, 50%). S+ was rewarded by a sucrose pellet if the rat nose poked in the reward magazine, OS− was not rewarded and nose poking in the reward magazine initiated a penalty inter-trial interval of 20 s. Rats learned to go immediately to the reward magazine after S+ (Go response) and to avoid nose poking into the reward magazine in response to OS− (NoGo response).

#### Phase 2: introduction of the Odor (O+) and discrimination between 3 stimuli

Phase 2 followed the same protocol as Phase 1, but now, three stimuli were randomly delivered: O+ (25% of the trials), S+ (25% of the trials) or the association OS− (50% of the trials). A sucrose pellet rewarded O+ and S+ if the rat nose poked in the reward magazine. OS− was not rewarded, nose poking in the reward magazine initiated a penalty inter-trial interval of 20 s.

Each rat performed daily conditioning sessions restricted to a 30 min period (about 60 trials).

### Behavior analysis

All the behavioral parameters were automatically recorded. For every trial, the latency between the stimulus port nose poke and the reward magazine nose poke was stored. Trials stopped 5 s after stimulations ended: if the rat did not nose poke in the reward magazine, the latency was set to 6.3 s (1.3 s of stim +5 s). Values were averaged by session (day) for each stimulus. A session was not taken into account if the number of trials initiated by the rat was lower than 5 (Fig. 1bi).

To normalize individual behavioral performances, 2 statistical indices were chosen to rate sessions as a function of the learning level: (i) Student t-test on latencies to select sessions where the rats discriminated OS− from O+ and S+ (p ≤ 0,05); (ii) *A*′ sensitivity index which discriminated between good hits and false alarms (*A*′ ≥ 0,75)^66^. The formula^67^ for *A*′ is

$$A^{\prime} =\{\begin{array}{c}\frac{3}{4}+\frac{H-F}{4}-F(1-H)\,{\rm{if}}\,F\le 0.5\le H\\ \frac{3}{4}+\frac{H-F}{4}-\frac{F}{4H}\,{\rm{if}}\,F\le H < 0.5\\ \frac{3}{4}+\frac{H-F}{4}-\frac{1-H}{4(1-F)}\,\mathrm{if}\,\,0.5 < F\le H\end{array}$$

*A*′ is a non-parametric estimate of the area under the Receiver Operating Characteristic (ROC) curve^68^, and is less sensitive to extreme false-alarm and hit rates than the classical d’^69^. *A*′ has been shown to provide the most suitable psychometric functions in go/no go experiments among several detectability indices^66,70^. We chose for *A*′ a threshold of 0.75, which is roughly equivalent to d’ = 1. According to these index, all the sessions of Phase 1 and 2 were sorted in 3 learning levels: Naïve (p ≥ 0,05 and *A*′ ≤ 0,75); Intermediate (p ≤ 0,05 or *A*′ ≥ 0,75, but not both) and Expert (p ≤ 0,05 and *A*′ ≥ 0,75).

### Electrophysiological recordings in behaving animals

The experimental arena was placed in a grounded Faraday cage. LFP signals were recorded during the entire behavioral training. Rats were connected to the recording device by a tether plugged into the implant connector in one end and a swiveling electrical connector that allowed free movements on the other end. Monopolar activity was acquired using a custom DasyLab (IOTECH, U.S.A) script driving an XCellAmp 64 amplifier (Dipsi, France) coupled with a DaqBoard 3000 USB system (IOTECH, U.S.A). The LFP signals and event markers (nose pokes) were amplified (X 2500), filtered (0.1–1000 Hz), digitized (sampling frequency, 2000 Hz) and stored on a computer.

### Signal selection

LFP signals were analyzed off-line with Matlab 2015a (Mathworks). A session was not taken into account if the number of trials initiated by the rat was lower than 5 (Fig. 1bi). For the other sessions, for each trial, signal analysis was performed on three task-related time intervals: one extending from [−3–2] s before stimulation onset and serving as a reference (Ref), another immediately before stimulation onset ([−1 0] s, Pre), and a third one immediately after stimulus onset ([0 1] s, Post). Only trials clean of artifacts in all these time intervals were kept, after careful visual examination of all trials.

### Time-frequency analysis

For each trial, a continuous Wavelet Transform using the Morlet wavelet was applied to the time signal (time resolution 0.5 ms, scale resolution 0.1; 57 scales between 2 and 100 Hz) then log-transformed. Values of this wavelet transform were judged significant if they were outside the range mean ± 2 SD computed on values from the Ref time interval at the same frequency.

For a given time and frequency, a Z-Score was computed as the wavelet power minus the average of power in the Ref period divided by the SD of the power in the Ref period. We computed an average Z-Score across all frequencies within the 3 frequency bands (Theta [8 12] Hz; Beta [15 35] Hz and Gamma [60 100] Hz). All quantifications on the time profile of Z-score were done on its maximum value taken over the time interval [300–600] ms after the nose poke, i.e. when the two stimuli were simultaneously present and before some artifacts due to the animal’s move induce factitious increase of the Z-score (especially in A1).

To assess multisensory interactions, we used a bootstrapping procedure^71^. In brief, we performed all possible linear summation of single unisensory (S+ and O+) trials recorded during the same session for a given animal, computed the above Z-Score and extracted the maximum of this Z-Score. We then randomly selected from all epoch combinations a number of maximum Z-Score equal to the number of OS− trials and computed their average. This sampling procedure was repeated 10000 times (bootstrap), which led to form a distribution against which average maximum Z-Score values for multisensory trials (OS−) may be statistically compared.

### Functional coupling

Directional flows between LFPs recorded in two regions were estimated by Directed Coherence (DCOH)^72^. The seminal work as well as previous papers^44,73,74^ detail the mathematical basis of DCOH and its rationale for biological signals. Briefly, DCOH is a spectral indicator (value between 0 and 1) that describes the frequency-specific correlation of two signals according to their temporal relationship. It estimates the fraction of the power spectral density of a signal Y explained by the spectral content of a signal X. Note that the DCOH includes a common input in its model which acts as a virtual reference allowing the decorrelation of the two random signals of interest. Mathematically, DCOH is a spectral transform of an autoregressive model with exogenous inputs (ARX) which linearly relate the current output Y(t) to a finite number of past inputs Y(t-k) (autoregressive part) and X(t-k) (exogenous part). In other words, we investigate how a LFP signal can be explained by a linear combination of its own past values as well as past values of another given signal. Computation details for ARX models are given in^75^. The order for the ARX model is often determined by information theory-based calculations (Akaike’s or Bayesian criterion^76,77^). In this study, since precise spectral intervals (frequency bands) were investigated, the model order has been chosen so that the ARX power spectra of X and Y were the most accurate to reflect peaks in X and Y power spectral density (PSD) estimated by fast Fourier transform (FFT). An order of 50 was chosen. DCOH was estimated for each trial with a frequency resolution of 1 Hz in the interval 0–50 Hz in the three time intervals of interest (Ref, Pre, Post).

### Prediction

Prediction of failure or success was estimated by the Matthew Correlation Coefficient (MCC = sqrt(Chi2_stat/n)^78^) computed from the confusion matrix obtained as follows: 104 variables comparing values across two time intervals were considered (including time variance of the signal, power in a given frequency band and DCOH between two areas in a given frequency band). Variables were normalized (averaged out and divided by their STD). For each variable, the average of values for failure and success subsets was computed. The value of a given trial was affected as a predicted success if it was closer to the average for success than failure and as a predicted failure otherwise. This resulted in a confusion matrix: rows correspond to the predicted responses, columns to the actual responses. Confusion matrices were also computed for groups of variables: each trial was then affected to failure if the vector of variable values was closer (using the Euclidean distance L2) to the vector value averaged over failure trials rather than over success trials. This allows making a prediction from multiple information instead of only one. Then, for either one variable or a group of variables, we performed a Chi2 test on the confusion matrix, which indicates if actual and predicted behavioral responses are significantly related. Matthew Correlation Coefficient is based on the Chi2 statistics and ranges from −1 (disagreement between actual and predicted results) to 0 for independence and 1 for perfect agreement corresponding to a diagonal confusion matrix.

### Statistical analysis

Was performed using ANOVA tests with two or three factors (among stimulation type, learning level of the animal and behavioral success of the trial). In that case, post-hoc analysis was performed with Student tests. A Bonferroni correction for multiple comparisons was systemically applied.

## Supplementary information

Dataset 1

## Data Availability

The datasets generated during the current study are available from the corresponding authors on reasonable request.
