# Supplementary material for: Multisensory learning between odor and sound enhances beta oscillations
Source: Sci Rep. 2019 Aug 2;9:11236. doi: 10.1038/s41598-019-47503-y (PMC6677763; doi:10.1038/s41598-019-47503-y)
Supplement: Supplementary file 1 — Dataset 1 [file 41598_2019_47503_MOESM1_ESM.pdf]

Supplementary information

Multisensory learning between odor and sound enhances beta oscillations

A. Gnaedinger[1], H. Gurden[1,2], B. Gourévitch[3,4,5,6 *], C. Martin[1,2,6 *]

[1]IMNC UMR 8165, Université Paris Sud, Université Paris Saclay CNRS Orsay F-91405, France.
[2]Unité de Biologie Fonctionnelle et Adaptative, UMR 8251, Université Paris Diderot, Sorbonne Paris Cité, CNRS F-75205, Paris, France.
[3]Unité de de Génétique et Physiologie de l'Audition, UMRS 1120, Institut Pasteur, INSERM F-75015 Paris, France.
[4]Sorbonne Universités, Université Pierre et Marie Curie, Complexité du Vivant, F-75015 Paris, France.
[5]CNRS, France.
[6]These authors contributed equally.

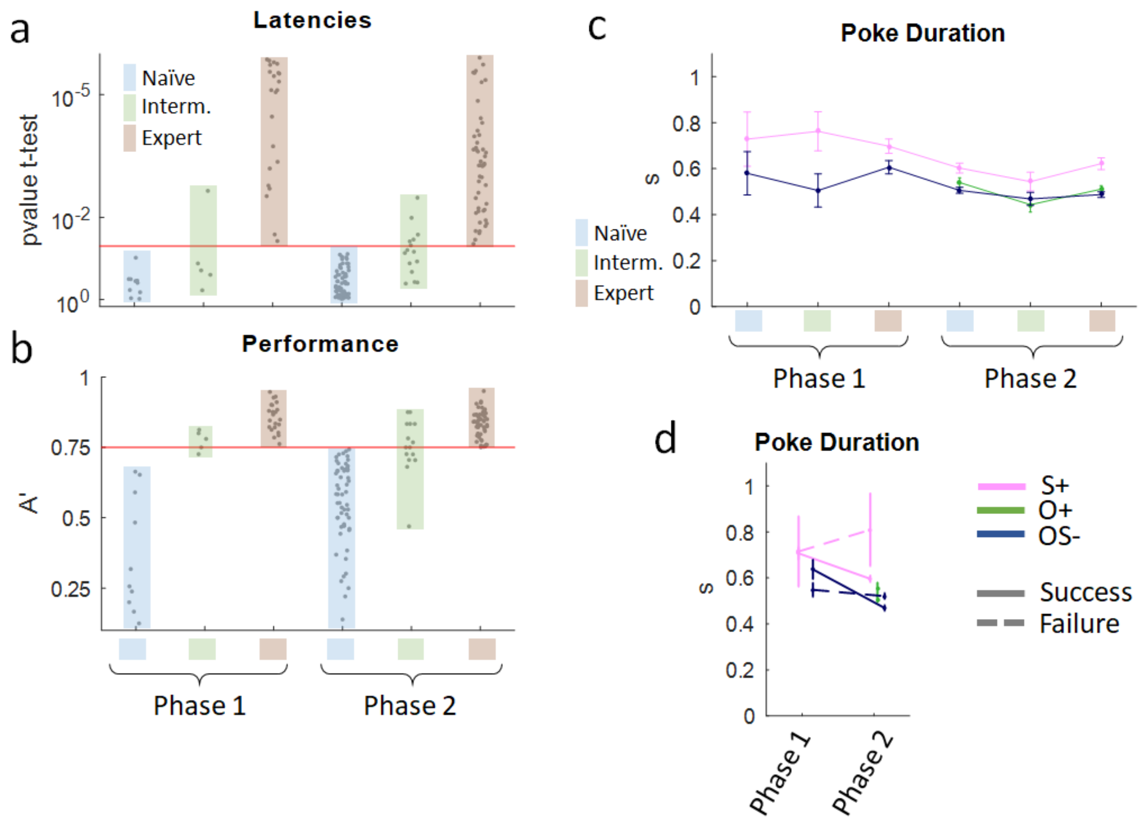

Supplementary Figure 1

Group results for behavioral performances (a) p-values for the Student t-test on latencies; each dot represents one session. The red line indicates the threshold chosen for the analysis. **(b)** Same than (a) for A' index values. **(c)** Average across learning levels of nose poke duration (in s) measured for each trial and each stimulus. **(d)** Effect of the behavioral output (success or failure) of the nosepoke duration (in s) for each stimulus in the two phases of the task.

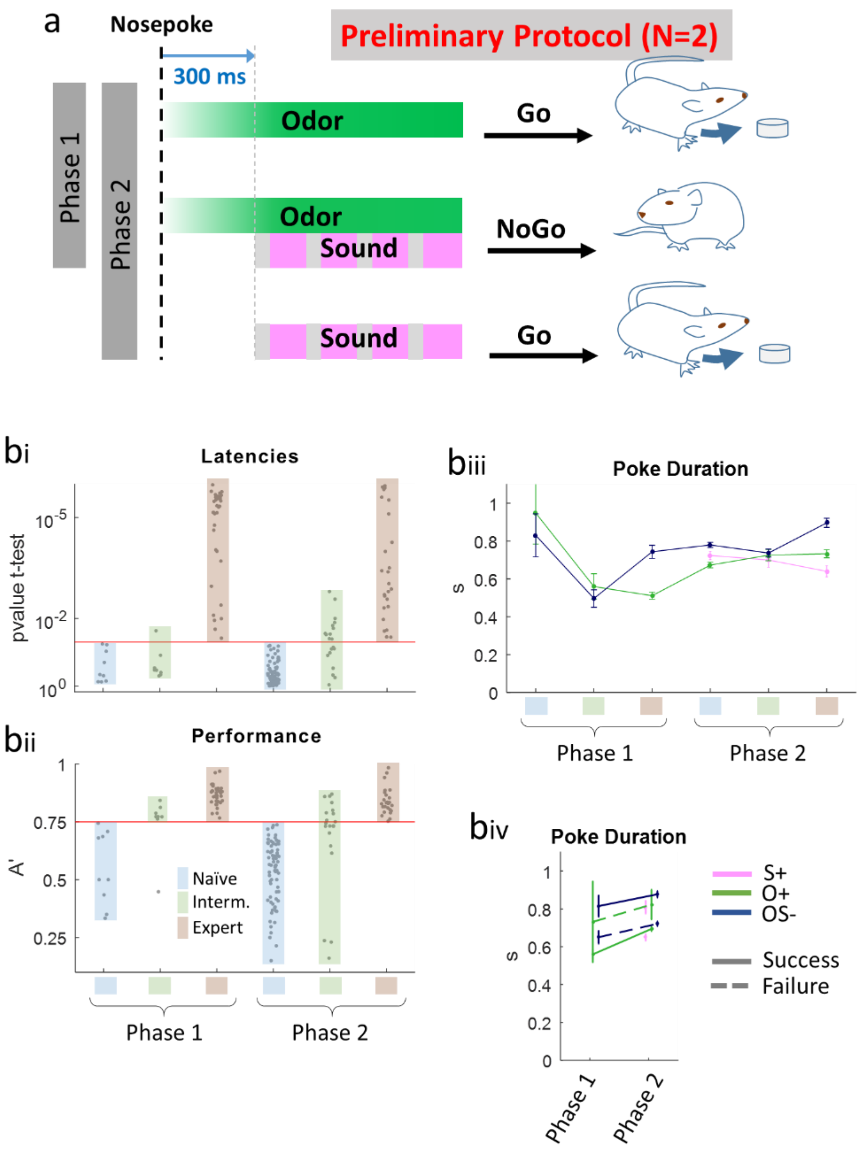

Supplementary Figure 2

Task design and behavioral performances for the odor and sound inverted protocol (a) Multisensory task design. Compared to the protocol described in Figure 1, S+ was replaced by O+ and conversely so that during Phase 1, two stimuli were randomly presented, odor (O+), rewarded by a sucrose pellet and odor/sound (OS-) not rewarded. In Phase 2, the third stimulus, sound (S+) rewarded by a sucrose pellet was added. Other parameters of the protocol are similar to that in Figure 1. This protocol was tested first at the beginning of the project and was proved efficient, albeit more difficult for the animals than the final protocol of the study. **(bi to biv)** similar to Supplementary Figure 1 for this preliminary protocol.

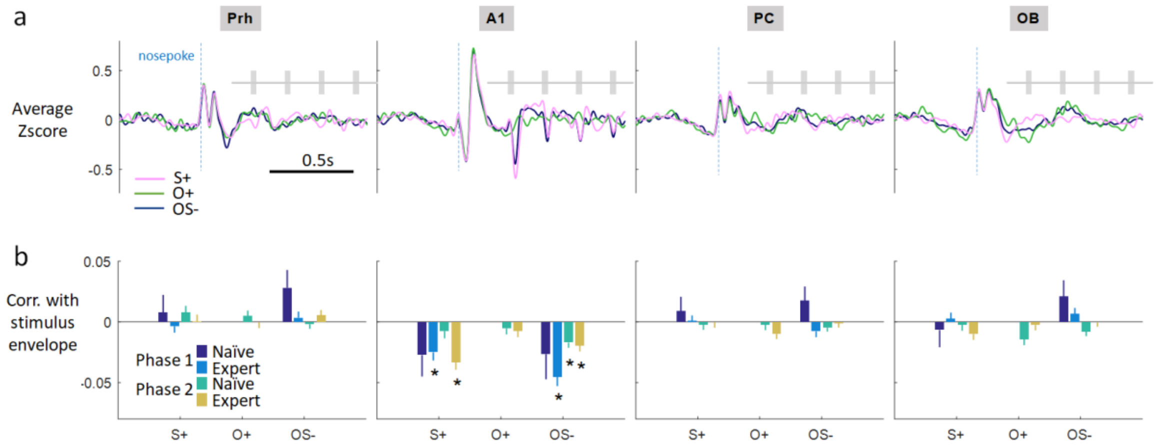

Supplementary Figure 3

Sensory evoked potentials activity during multisensory learning (a) Z-score normalized average of LFP signal's amplitude between -0.5 and 1 s relative to the nose poke in the stimulus port in the 4 brain areas and for the 3 stimuli. White noise bursts of the auditory stimulus are represented as grey vertical bars. In all the brain regions, the nose poke itself (which triggers the stimulation) evokes a long lasting potential modulation including positive and negative components. The negative deflection was only recorded in A1 where it was of high amplitude and identical for the 3 stimuli. This potential is time locked to -and thus likely evoked by- a click sound produced by the behavioral apparatus. On the contrary, a positive deflection could be seen in OB, PC and Prh and was similar between stimuli. This is of unclear origin, it could be related to sniffing. **(b)** Average of the correlation between the evoked activity of each trial (grouped by level, brain area and stimulus) and the temporal envelope of the auditory stimulus. Student tests are used here (p-value threshold is 0.05/10).

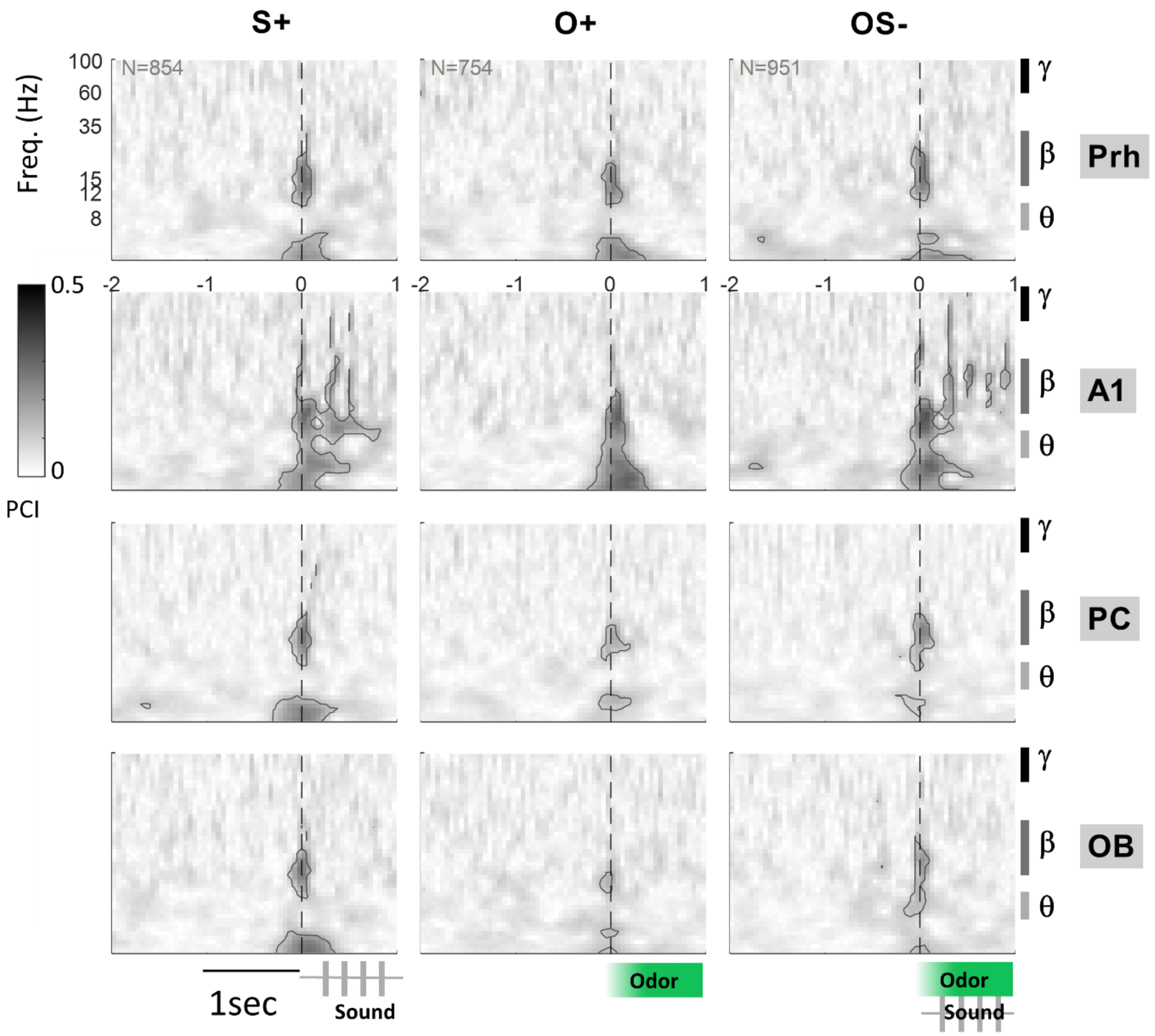

Supplementary Figure 4

Phase resetting We quantified the Phase Concentration Index (PCI) across trials [1–4]. Briefly, we extracted the quantity exp(*i***phi*) with *i* being the imaginary number and *phi* being the phase of the complex result of the above wavelet transform for each time point and frequency within a given trial. Such quantity (of modulus 1) was then averaged across trials and its modulus is called PCI. PCI ranges from 0 (random set of phases) to 1 (perfect phase alignment across trials). Phase synchronization index (grey intensity) is plotted here as time-frequency maps for the 3 stimuli (columns) and the 4 brain areas (rows). Dotted vertical line at time 0 represents the nose poke. Significance of PCI was obtained through a Rayleigh test [5] for each time-frequency « pixel ». As Bonferroni correction is known to be somewhat conservative if there are a large number of tests [6], we set a correction factor of 100 for the alpha risk of each test.

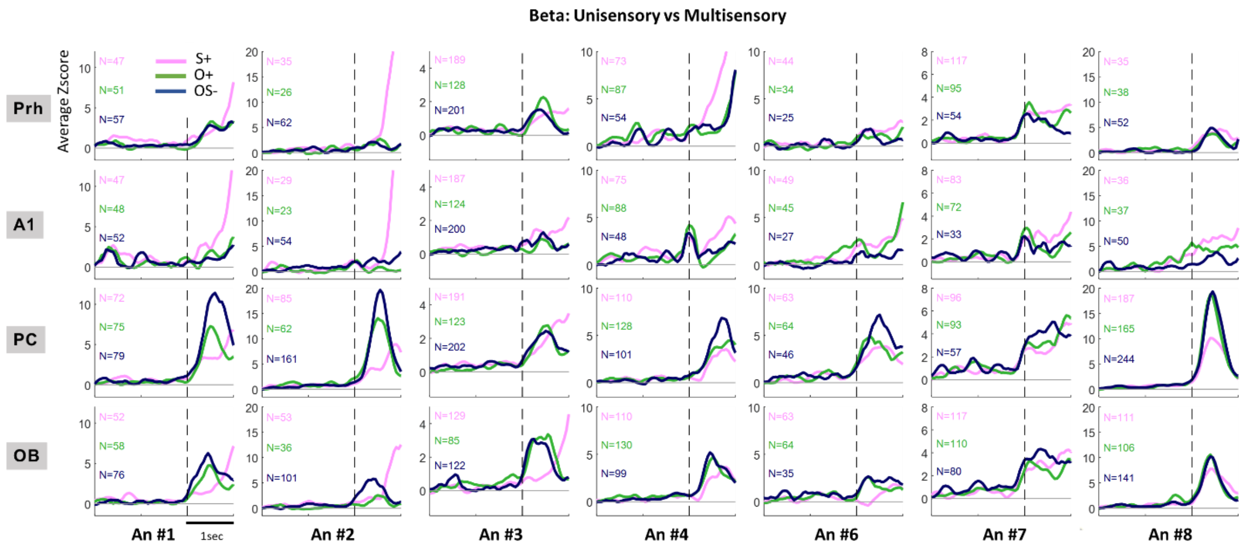

Supplementary Figure 5

Power of oscillations in beta (15-35 Hz) in uni- and multimodal conditions for each animal. Profile of z-score normalized power in beta (15-35 Hz) and gamma (60-100 Hz) bands as a function of time, from –2 s to 1 s relative to the nose poke (dotted vertical line). Power is represented for each stimulus as a different color line. Only success trials of Phase 2 are selected. The number of trials is indicated for each condition.

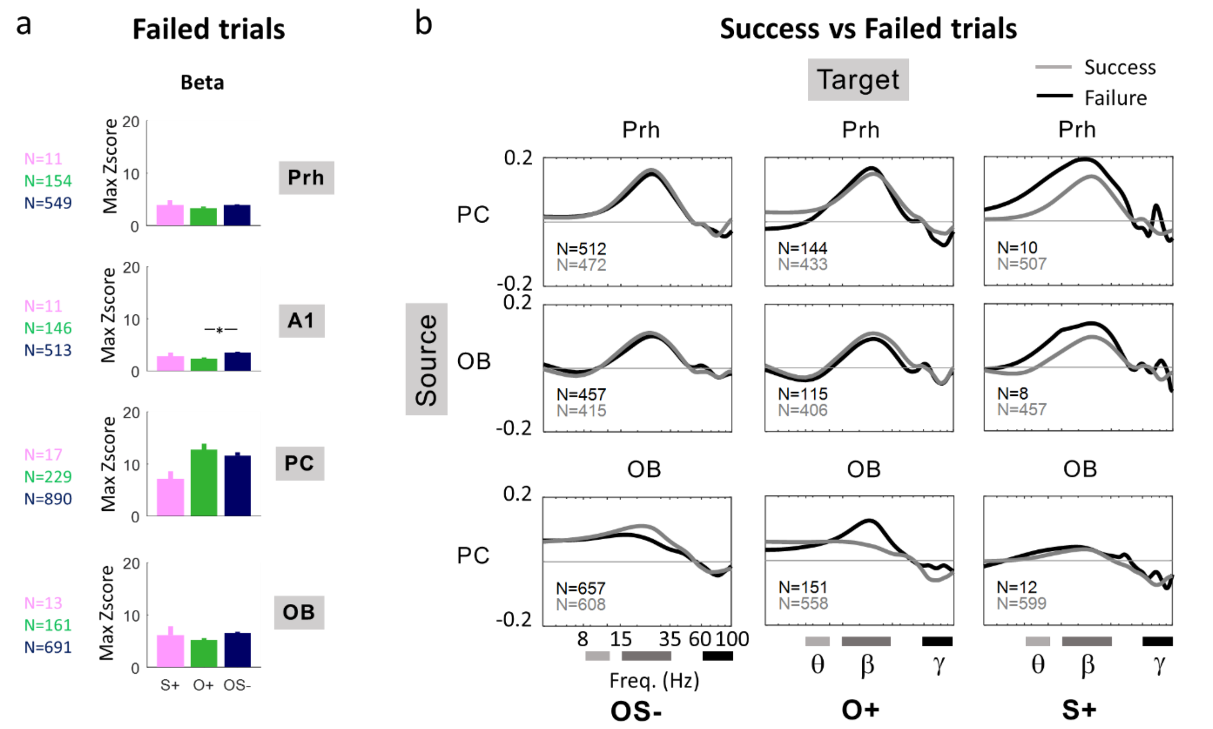

Supplementary Figure 6

Beta oscillations compared between correct and incorrect responses. (a) Maximum averaged Zscore in the beta band during stimulus sampling (calculated in the time range [0.3 0.6] s) for incorrect responses. Data are displayed as mean +/-s.e.m.. Same color code as in Fig. 3a) *p<0.05/3 (Bonferroni on testing pairs). **(b)** Flows between pairs of structures (source in line, target structure in column) estimated as the difference of DCOH between the stimulation period ([0 1] s) and the Pre period ([-1 0] s). Success (gray) and failed trials (black) of Phase 2 are compared for the three stimuli.

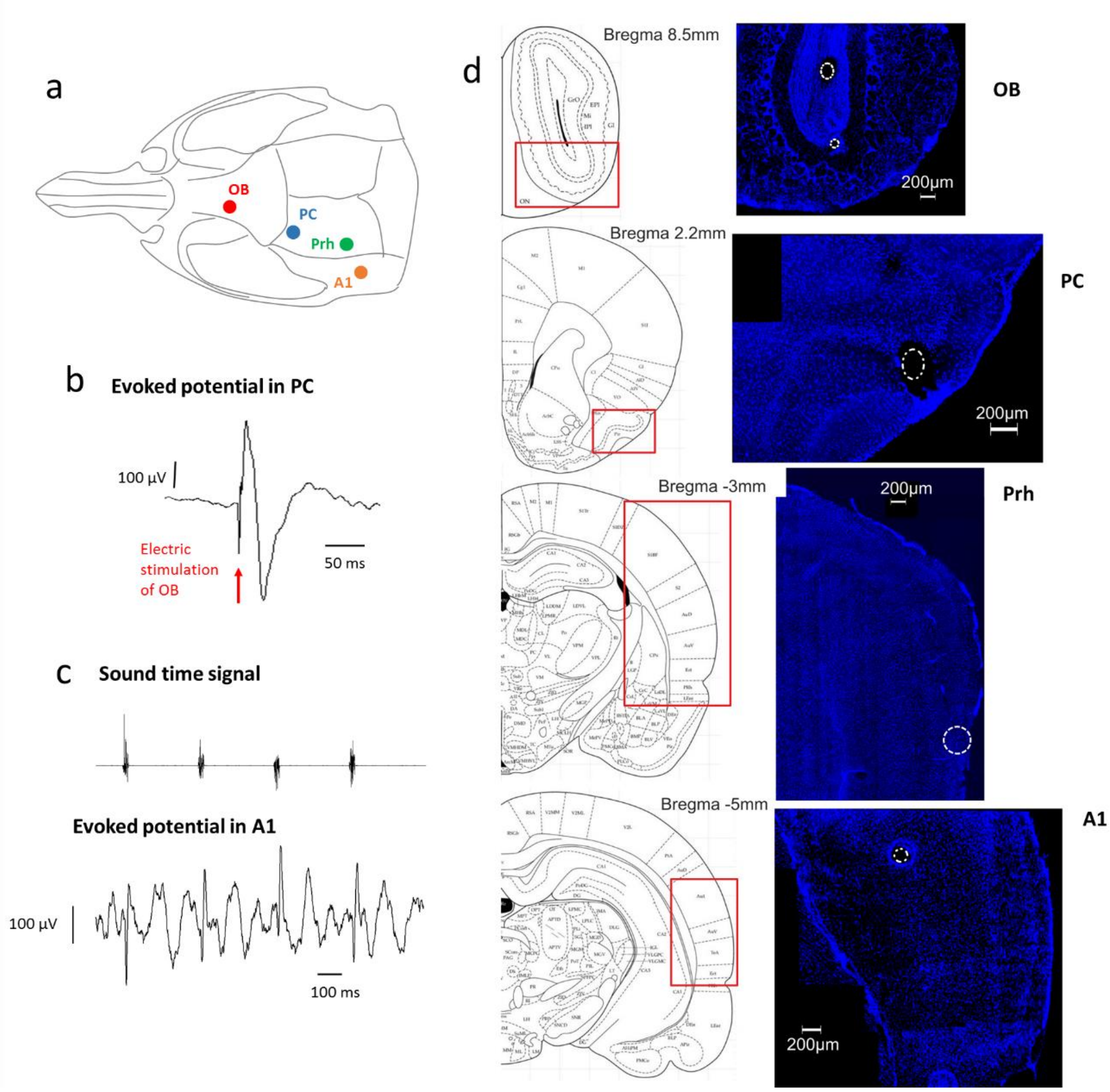

Supplementary Figure 7

Anatomical and functional validation of implantations. (a). Stereotaxic positions of recording electrodes. **(b).** In the PC, the depth of the electrode was set using the field-potential profile evoked in response to electrical stimulation of the bipolar OB electrodes. **(c)**. In A1 the position of the electrode was set using the field-potential profile evoked in response to a sequence of white noise. **(d)**. Brain sections with DAPI labeled cellular nuclei showing example of electrodes placement. The locations of the electrode tips in the brain were marked by passing a current (250 mA, 5x100ms) through them. Drawings modified with permission from [7], pictures built using the Fiji stitching plugin[8]

References

1. Delorme, A. & Makeig, S. EEGLAB: an open source toolbox for analysis of single-trial EEG dynamics including independent component analysis. *J. Neurosci. Methods* **134**, 9–21 (2004).
2. Makeig, S. *et al.* Dynamic brain sources of visual evoked responses. *Science* **295**, 690–694 (2002).
3. Mercier, M. R. *et al.* Neuro-oscillatory phase alignment drives speeded multisensory response times: an electro-corticographic investigation. *J. Neurosci. Off. J. Soc. Neurosci.* **35**, 8546–8557 (2015).
4. Tallon-Baudry, C., Bertrand, O., Delpuech, C. & Pernier, J. Stimulus specificity of phase-locked and non-phase-locked 40 Hz visual responses in human. *J. Neurosci. Off. J. Soc. Neurosci.* **16**, 4240–4249 (1996).
5. Mardia, K. V. *Statistics of directional data*. (Academic Press, 1972).
6. Bland, J. M. & Altman, D. G. Multiple significance tests: the Bonferroni method. *BMJ* **310**, 170 (1995).
7. The Rat Brain in Stereotaxic Coordinates, 6th Edition | George Paxinos, Charles Watson | ISBN 9780125476126. Available at: http://store.elsevier.com/The-Rat-Brain-in-Stereotaxic-Coordinates/George-Paxinos/isbn-9780125476126/. (Accessed: 13th September 2016)
8. Preibisch, S., Saalfeld, S. & Tomancak, P. Globally optimal stitching of tiled 3D microscopic image acquisitions. *Bioinformatics* **25**, 1463–1465 (2009).
